# Multimodal Ultrasound Manifestations of a Central Intraductal Papilloma at the Nipple and Its Causes of Misdiagnosis: A Case Report

**DOI:** 10.7759/cureus.98355

**Published:** 2025-12-03

**Authors:** Chen Mingrui, Xu XiaoHong, Zhong XinRong

**Affiliations:** 1 Ultrasound Medicine, Affiliated Hospital of Guangdong Medical University, Zhanjiang, CHN

**Keywords:** contrast-enhanced ultrasound, differential diagnosis, intraductal papilloma of the breast, pathological diagnosis, ultrasound examination

## Abstract

Intraductal papillomas (IPs) are tumors arising from the ductal epithelial cells of the breast. Common symptoms associated with IPs include serous or serosanguineous nipple discharge and palpable masses. We present a case involving a 59-year-old female patient who experienced papillary lesions and nipple discharge for 20 years. Diagnostic imaging for this patient included mammography, conventional ultrasound, and contrast-enhanced ultrasound, which revealed an irregular and poorly defined mass at the right nipple. The ultrasound findings raised the possibility of Paget's disease of the breast, while a biopsy of the right papillary neoplasm confirmed the diagnosis of an IP. The presentation of an IP as a neoplasm is uncommon. This article primarily discusses rare cases and examines multimodal ultrasound manifestations to enhance the predictive accuracy of diagnosing both benign and malignant breast lesions, thereby reducing the rate of misdiagnosis.

## Introduction

Intraductal papillomas (IPs) are benign tumors located within the mammary ducts. The abnormal proliferation of ductal epithelial cells contributes to tumor growth [1], and they can manifest in women across all age groups [2]. Histologically, IPs are characterized by a fibrovascular core enveloped by epithelial and myoepithelial cells [2]. Given their association with atypical ductal carcinoma in situ (DCIS) and breast cancer, these lesions are classified as high-risk precursor lesions [1]. Peripheral IPs are the most prevalent variant, typically situated in the periphery of any breast quadrant. In contrast, central IPs are generally located within the larger ducts of the subareolar region, often presenting as a solitary mass [1]. Occasionally, IP masses can be palpated. When the delicate epithelial cells within these masses rupture and bleed, they may result in bloody secretions from the papillae. Compared to the peripheral type, central IPs are more likely to induce spontaneous nipple discharge. The peripheral variant usually manifests as multiple lesions and infrequently causes nipple discharge [3]. Isolated IPs are commonly found in the central or regional area behind the areola [1], and the occurrence of papillary neoplasms associated with it is relatively uncommon. Currently, there are limited reports addressing related special manifestations. The detection of IPs primarily relies on conventional ultrasound examination. Differential diagnosis can be achieved through fine-needle aspiration biopsy, mammography, MRI, and other modalities; however, reports focusing solely on differential diagnosis via contrast-enhanced ultrasound are also scarce. This report presents the multimodal ultrasound manifestations of a case of IP of the breast with a unique location, as confirmed by pathology.

## Case presentation

**Figure 1:**
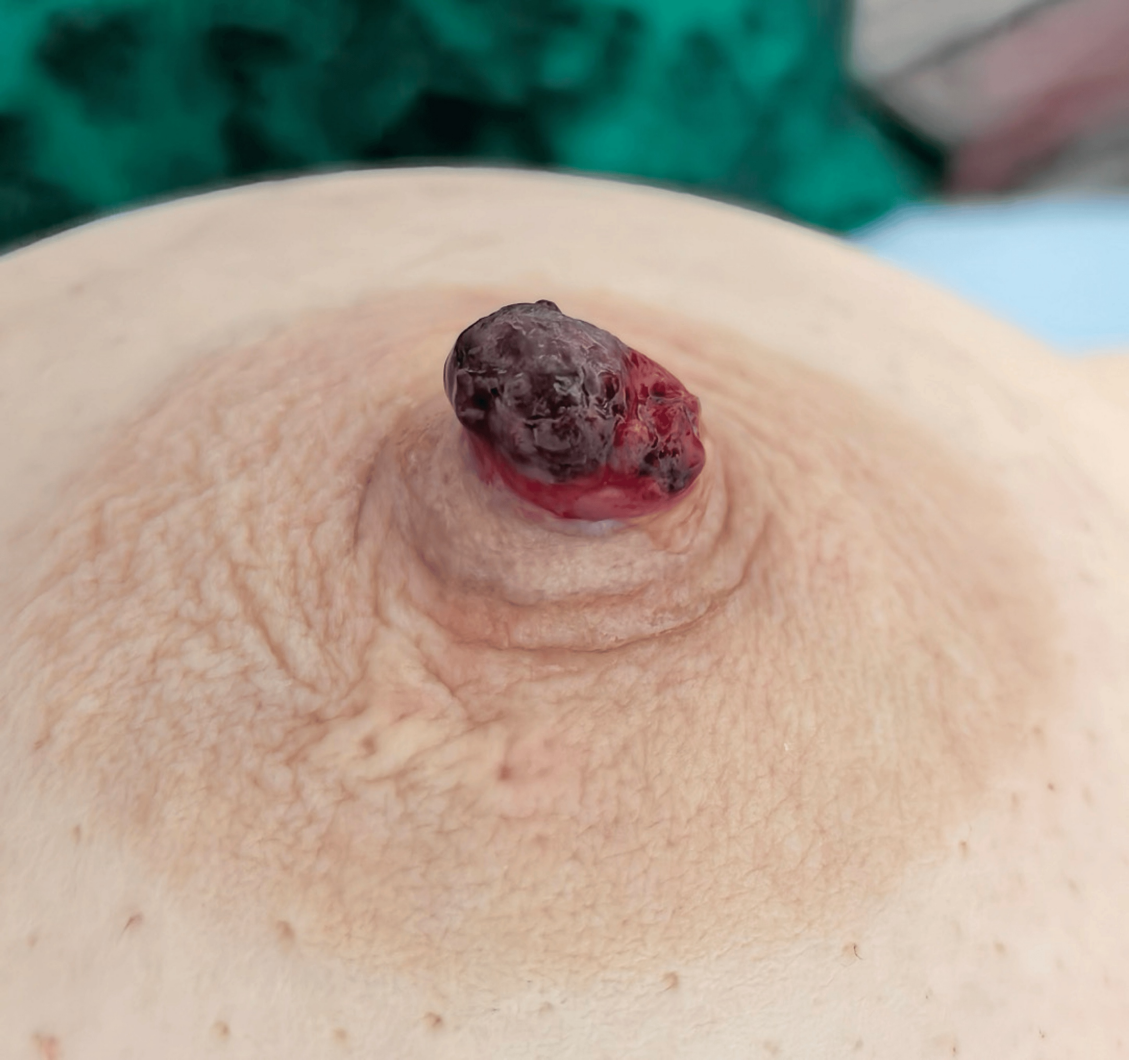
A side view of the right nipple The blood, granulation tissue and scab complex look like a neoplasm to the naked eye.

A 59-year-old female patient presented to our hospital for treatment three days after the identification of papillary neoplasms associated with nipple discharge that had persisted for 20 years. Physical examination revealed cauliflower-like raised lesions at the nipples, characterized by red scabs of varying shades on the surface (as depicted in Figure 1). The lesion protruding from the nipple measured approximately 10 mm × 10 mm and exhibited limited mobility. No abnormal changes were noted in the surrounding tissues. The patient reported that over the 20-year duration, the skin at the nipples occasionally experienced itching, redness, and swelling. Following episodes of scratching and ulceration, scabs would form and subsequently heal, providing temporary relief. This cycle of symptoms recurred repeatedly.

**Figure 2:**
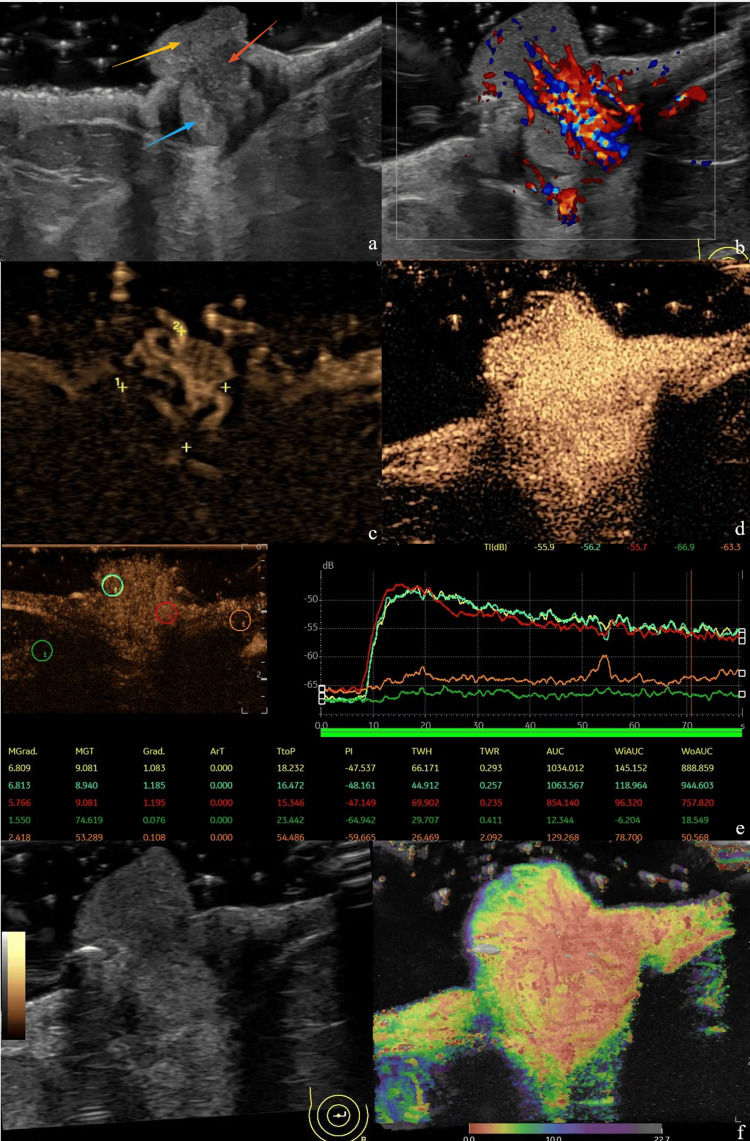
The ultrasound image showing the cross-section of the subpapillary lesion a. Two-dimensional ultrasound image: It can be seen that the slightly lower echo zone, the isoechoic zone and the neoplasms at the nipple seem to form a whole. The red arrows indicate the slightly lower echo zone, the blue arrows indicate the isoechoic zone, and the yellow arrows indicate the protruding papillae. b. CDFI: The lesion at the right nipple is rich in bundle blood flow signals, with Alder grade 3. c. B-Flow condition: The longitudinal section of the nipple reveals a quasi-circular isoechoic region, suggesting that certain sections exhibit a clear boundary between this area and the neoplasms located at the nipple. At the ninth second of contrast-enhanced ultrasound, a concentric high enhancement was observed, and the lesion area increased following enhancement. d-e. CEUS: At the ninth second after the contrast agent was injected through the anterior elbow vein in contrast-enhanced ultrasound, the lesion area expanded compared with the conventional ultrasound range. The TIC curve analysis shows that the enhancement amplitude and pattern of the protruding part of the nipple and the lesion tissue beneath the nipple are comparable. f. MVI: Ultra-micro blood flow imaging using the Sonazoid contrast agent reveals that micro-blood flow is directed from the peripheral tissue toward the center of the lesion, subsequently dispersing from the center to the periphery. Additionally, the curvature of the blood vessels remains regular.

Ultrasound examination revealed a nearly circular isoechoic nodule located behind the right nipple, characterized by a well-defined boundary. The nodule extended from the area behind the right nipple to the nipple's surface, measuring approximately 10 mm × 9 mm (Figure 2a). Color Doppler flow imaging indicated an abnormal richness of blood supply within the nodule, which was nearly entirely filled with blood vessels, classified as Alder grade III (Figure 2b). Under B-Flow conditions, a substantial blood supply at the lesion site was observable (Figure 2c). Contrast-enhanced ultrasound (CEUS) scanning of the lesion demonstrated enhancement beginning nine seconds post-injection. The area of enhancement was broader compared to two-dimensional imaging, exhibiting uniform and high enhancement throughout the observation period. The degree of enhancement at the lesion site was comparable to that of the papillary tissue, with peak enhancement occurring more rapidly than in the areola tissue, though the boundaries remained unclear (Figures 2d-2e). Microvascular imaging (MVI) reveals evidence of penetrating blood flow, initially observed at the periphery of the lesion before extending to the nipple and the lesion itself. A significant vascular supply is present within the lesion, radiating outward toward the periphery and the nipple (Figure 2f). Following a multimodal ultrasound examination, the nodule located behind the right nipple was classified as BI-RADS 4a, raising suspicion for Paget disease of the breast.

**Figure 3:**
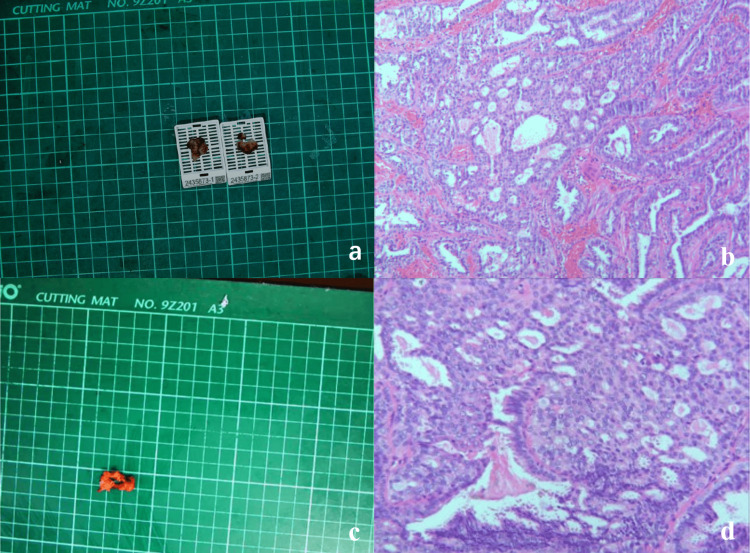
a. Right papillary neoplasm incision specimen: dimensions 15 mm × 15 mm × 7 mm, with a cut surface that is grayish-white and grayish-red, exhibiting a medium texture. b. Biopsy histopathological image of the right papilloma: this image is consistent with intraductal papilloma, showing partial common hyperplasia of the ductal epithelium and columnar cell hyperplasia. c. Surgical incision of a mass specimen beneath the right nipple: dimensions 22 mm × 19 mm × 5 mm. A nodule is visible on the section, with a maximum diameter of approximately 8 mm. It appears grayish-white or grayish-yellow, has a medium texture, and presents an unclear boundary. d. Biopsy histopathological image of the mass beneath the right papilla: this image is consistent with intraductal papilloma of the breast, displaying common hyperplasia and columnar cell hyperplasia of the local ductal epithelium, along with neutrophil infiltration in the focal stroma

A biopsy was obtained from the local tissue of the neoplasm located at the right papilla, measuring approximately 15mm × 15mm × 7mm. The cross-section exhibited a grayish-white or grayish-red coloration and demonstrated a medium texture. Pathological analysis confirmed the diagnosis of an IP. Immunohistochemical findings revealed CK5/6 (mottled +), SOX-10 (mottled +), myoepithelial cells (positive), and estrogen receptor (ER) positivity (dappled), with Ki67 at approximately 5%. Following evaluation, surgical indications were deemed appropriate, leading to the performance of a right breast mass resection and papillary plasty. Postoperative pathological results reaffirmed the diagnosis of IP of the breast, characterized by common hyperplasia and columnar cell hyperplasia of the local ductal epithelium, along with neutrophil infiltration in the focal stroma (Figure 3).

## Discussion

CEUS revealed that the lesion in this case exhibited uniform and pronounced centripetal enhancement. Following enhancement, the area of the lesion expanded, displaying an indistinct boundary with the surrounding papillary skin and evidence of mutual fusion. This observation raises the possibility of malignancy or inflammatory infiltration. The blood supply characteristics in both scenarios are notably similar, which can easily lead to confusion among clinicians.

Tumor growth is contingent upon the formation of vascular networks. High microvessel density correlates with a poor prognosis in cases of high-grade, aggressive breast cancer [4]. Although the Adler classification is the most widely employed criterion for vascular assessment, Adler et al. [5] demonstrated that its ability to differentiate between healthy breast tissue and cancerous tissue is limited. Consequently, there is a need for more sensitive methods to detect microvascular blood flow associated with cancer.

Recent research, in conjunction with the novel angiogenesis hypothesis [4], assigns "vascular imaging scores" using a three-factor system that evaluates ductal lesions and predicts malignant tumors based on the number, shape/complexity, and distribution pattern of blood vessels. This scoring system assesses three dimensions: the quantity of blood vessels (0-4), their distribution (0-3), and their morphology (0-3), yielding a maximum score of 10 points. The optimal cut-off value for the total vascular score indicative of malignant tumors is 8 points [6]. Among the dilated catheters, the scoring system assigns 1 point for 1-2 vessels, 2 points for 3-4 vessels, 3 points for 5-7 vessels, and 4 points for more than eight vessels. Vascular distribution is categorized as periductal (1 point), intraductal (2 points), or both (3 points). Vascular morphology (complexity) is classified as follows: punctate (1 point), parallel to the catheter in a straight line without crossing the vessel wall (2 points), or penetrating and branching across the vessel wall (3 points). In this instance, microblood flow from the lesion manifests as a penetrating band-like area of blood supply that extends from the periphery of the lesion inward, creating a circular isoechoic region. Under reperfusion conditions, this isoechoic region gradually expands toward the papillary skin from the central point. The blood supply characteristics can be rated at 8 points within the three-factor scoring system [4,6], which aligns precisely with the standard dividing line for positive malignant tumors.

Based on the biological behavior of the lesion in this case, as determined by comprehensive CEUS and MVI model examinations, it is posited that the initial primary lesion may have originated from a circular, echogenic area beneath the nipples. Over time, as the disease progressed, it gradually extended to the skin of the nipples, resulting in the formation of the papillary neoplasm observable in clinical practice. However, the tissue density of the newly developed lesions does not align with that of the original lesions.

The misdiagnosis of Paget's disease in this case can be attributed to several factors. First, the blood supply within the lesion is abnormally abundant. Second, the lesion affects the nipples and the surrounding area, with the cauliflower-like growths at the nipples being particularly misleading. Third, the persistent itching and scabbing over a 20-year period resemble the clinical manifestations of Paget's disease of the breast. Previous studies have indicated that some patients with Paget's disease of the breast present with normal preoperative imaging, yet postoperative pathology may still reveal small foci of invasive cancer, highlighting a significant false negative rate associated with ultrasound and mammography examinations [7]. Consequently, when clinical manifestations are similar and the mass evaluated using the three-factor scoring method lies on the standard dividing line for malignant tumors, heightened vigilance is warranted. Postoperative pathology indicates that inflammatory changes, characterized by local neutrophil infiltration, accompany IP, which aligns with the underlying principles of multimodal ultrasound manifestations.

## Conclusions

Ultrasound examination poses no radiation risk and offers convenience through real-time dynamic observation. The contrast agent utilized in CEUS is also devoid of liver or kidney toxicity, representing a distinct advantage of ultrasound over other imaging modalities. In comparison to traditional ultrasound, multimodal ultrasound provides a more comprehensive assessment of lesions. CEUS and MVI can elucidate the source of blood supply, the direction of blood flow, and the structural characteristics of neovascularization within the lesion. In cases of breast tumor screening where differentiation between benign and malignant types is challenging, the three-factor scoring rule remains applicable for evaluating tumor characteristics. This approach significantly reduces the likelihood of misdiagnosis and effectively guides clinical decision-making in selecting appropriate treatment strategies.
